# Modeling climate change impacts and predicting future vulnerability in the Mount Kenya forest ecosystem using remote sensing and machine learning

**DOI:** 10.1007/s10661-025-14089-0

**Published:** 2025-05-06

**Authors:** Terry Amolo Otieno, Loventa Anyango Otieno, Brian Rotich, Katharina Löhr, Harison Kiplagat Kipkulei

**Affiliations:** 1 Department of Geomatic Engineering and Geospatial Information Systems, Jomo Kenyatta University of Agriculture and Technology (JKUAT), P.O. Box, Nairobi, 62000 00200 Kenya; 2 Faculty of Environmental Studies and Resources Development, Chuka University, P.O. Box 109–60400, Chuka, Kenya; 3 Faculty of Forest and Environment, Eberswalde University for Sustainable Development (HNEE), Alfred-Moeller-Str. 1, 16225 Eberswalde, Germany; 4 Center for Climate Resilience, University of Augsburg, Universitätsstraße 12, 86159 Augsburg, Germany

**Keywords:** Forests, Vulnerability, Climate modeling, Sustainable management

## Abstract

The Mount Kenya forest ecosystem (MKFE), a crucial biodiversity hotspot and one of Kenya’s key water towers, is increasingly threatened by climate change, putting its ecological integrity and vital ecosystem services at risk. Understanding the interactions between climate extremes and forest dynamics is essential for conservation planning, especially in the Mount Kenya Forest Ecosystem (MKFE), where rising temperatures and erratic rainfall are altering vegetation patterns, reducing forest resilience, and threatening both biodiversity and water security. This study integrates remote sensing and machine learning to assess historical vegetation changes and predict areas at risk in the future. Landsat imagery from 2000 to 2020 was used to derive vegetation indices comprising the Normalized Difference Vegetation Index (NDVI), Enhanced Vegetation Index (EVI), Soil-Adjusted Vegetation Index (SAVI), and Bare Soil Index (BSI). Climate variables, including extreme precipitation and temperature indices, were extracted from CHIRPS and ERA5 datasets. Machine learning models, including Random Forest (RF), XGBoost, and Support Vector Machines (SVM), were trained to assess climate-vegetation relationships and predict future vegetation dynamics under the SSP245 climate scenario using Coupled Model Intercomparison Project Phase 6 (CMIP6) downscaled projections. The RF model achieved high accuracy (*R*^2^ = 0.82, RMSE = 0.15) in predicting the dynamics of vegetation conditions. Model projections show a 49–55% decline in EVI across forest areas by 2040, with the most pronounced losses likely in lower montane zones, which are more sensitive to climate-induced vegetation stress. Results emphasize the critical role of precipitation in sustaining forest health and highlight the urgent need for adaptive management strategies, including afforestation, sustainable land-use planning, and policy-driven conservation efforts. This study provides a scalable framework for modelling climate impacts on forest ecosystems globally and offers actionable insights for policymakers.

## Introduction

Mount Kenya, standing at 5199 m, is Kenya’s highest mountain and a UNESCO World Heritage Site, recognized for its diverse ecosystems that range from montane forests to afro-alpine zones (Nature Kenya, 2019). These forests play a critical role in carbon sequestration, water regulation, and biodiversity conservation, supporting endemic species such as the Mountain Bongo (*Tragelaphus eurycerus isaaci*) and the Mount Kenya Bush Viper (*Atheris desaixi*). Additionally, the forest ecosystem serves as a major water tower, supplying over 40% of Kenya’s freshwater resources, which are essential for hydroelectric power, agriculture, and urban consumption (Nyongesa & Vacik, 2019).

Despite its ecological significance, mount Kenya’s forest is increasingly vulnerable to climate change and anthropogenic pressures. Rising temperatures and shifting precipitation patterns have altered forest dynamics, vegetation composition, and species distributions, leading to reduced forest resilience (Downing et al., 2024). Moreover, human activities such as illegal logging, agricultural encroachment, and unregulated grazing exacerbate forest degradation, further threatening ecosystem stability (Ndalila et al., 2024; Rotich et al., 2025).

Climate change is driving extreme weather events, including increased temperatures, erratic rainfall patterns, and prolonged droughts, which have a direct impact on forest health. Recent studies predict widespread vegetation shifts across Kenya, with increased dryland expansion at the expense of montane forests (Parracciani et al., 2023). Research in the East African montane regions has demonstrated that high-elevation ecosystems are particularly sensitive to warming trends, with species migrating downslope in search of favorable conditions (Jia et al., 2024). This migration disrupts existing ecological gradients, leading to potential species loss and altered carbon cycling. Furthermore, the rapid retreat of Mount Kenya’s glaciers, primarily attributed to rising temperatures, has intensified hydrological imbalances, reducing water availability for both human and ecological needs (Chen et al., 2018; Mwaniki et al., 2018). As a result, lower montane forests, which depend on stable moisture conditions, are experiencing increased vulnerability to drought stress and biomass loss (Terschanski et al., 2024). Climate change impact on forest ecosystems and biodiversity has been conducted in the East Africa region to analyze the response of vegetation to multiple climate change variables (John et al., 2020; Parracciani et al., 2023).

Remote sensing provides an effective and scalable approach to monitoring forest dynamics by utilizing vegetation indices derived from satellite imagery. The Normalized Difference Vegetation Index (NDVI), Enhanced Vegetation Index (EVI), Soil-Adjusted Vegetation Index (SAVI), and Bare Soil Index (BSI) are commonly employed to assess vegetation health, land degradation, and forest cover changes over time (Rodriguez-Esparragón et al., 2024). Advancements in machine learning techniques have further enhanced the ability to analyze climate-vegetation interactions and predict future ecological trends. Random Forest (RF), Extreme Gradient Boosting (XGBoost), and Support Vector Machines (SVM) are widely used models for evaluating non-linear relationships between climate variables and vegetation indices (Anees et al., 2024). Furthermore, the models can be integrated with future climate modelling data to allow for the prediction of climate-induced forest changes. The evaluation of future conditions is conducted relative to historical satellite observations to analyze the impact of change in climate variables on forest conditions. Climatic scenarios such as the coupled model intercomparison project phase 6 (CMIP6) and shared socioeconomic pathways (SSP) provide a robust framework for assessing long-term forest vulnerability under moderate emission pathways (Eyring et al., 2016).

Forest ecosystem monitoring and analysis of climate change impacts have been addressed in the literature. For instance, Taccoen et al. (2022) assessed the effects of temperature and rainfall changes on background tree mortality rates in France using the logistic regression model. Gunn et al. (2019) evaluated degradation in a North American temperate forest by quantifying recent trends in forest stocking components and the role of various management and climatic variables. Furthermore, Abera et al. (2024) combined temperature and remote sensing indices to evaluate the impact of deforestation on climate change effects in African montane forests. Other studies have also been conducted in Asia and Australia to examine the impact of climate change on terrestrial flora (Hoffmann et al., 2019; Wu et al., 2010).

Despite significant research on the impacts of climate change on forest ecosystems globally, key knowledge gaps remain. For instance, there is limited predictive modelling of climate-induced vegetation changes in montane ecosystems, limited evidence on ecosystem monitoring with the integration of machine learning and remote sensing data, vulnerability assessments of montane ecosystems under future climates, and limited spatial-explicit mapping of vulnerable forest zones under future climate scenarios. Similarly, scientific studies have primarily focused on mapping vegetation characteristics and land-use change dynamics in the regional water towers and montane ecosystems. Additionally, other studies have examined the effects of forest fires on vegetation characteristics (Henry et al., 2019; Willkomm et al., 2016). Moreover, these studies primarily focus on historical assessments rather than forecasting future vulnerabilities under climate scenarios. Therefore, this study bridges these gaps by combining multi-temporal satellite imagery, climate data, and advanced machine learning models to analyze and predict the future vulnerability of forest ecosystem conditions, focusing on the MKFE. The findings will inform adaptive forest management strategies, conservation planning, and climate mitigation policies aimed at preserving MKFE. Specifically, the study aims to assess the impact of climate change on Mount Kenya’s forest dynamics and predict future vulnerable areas. Thus, the objectives of the study are to (i) quantify forest conditions using satellite vegetation indices (NDVI, EVI, SAVI, BSI) from 2000 to 2020, (ii) analyze correlations between climate variables and vegetation indices to determine key drivers of forest conditions decline, and (iii) model and predict future vegetation conditions using machine learning under the SSP245 climate scenario. By leveraging remote sensing, climate modeling, and machine learning, this study provides a scalable framework for assessing climate risks on forest ecosystems globally while offering data-driven insights for policymakers and conservationists.

## Materials and methods

### Study area

**Fig. 1:**
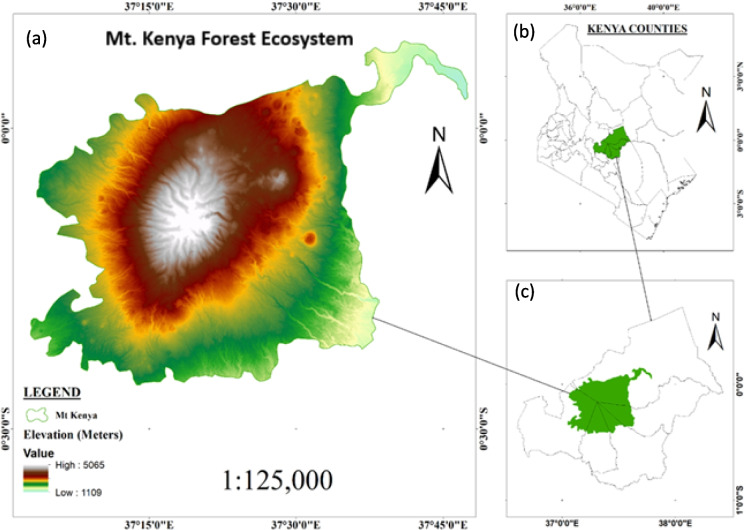
Study area map: **a** Mt. Kenya Forest Ecosystem with a digital elevation model superimposed on the extents, **b** location of Mount Kenya Ecosystem in Kenya, **c** location of Mount Kenya Ecosystem within the central Kenya counties

Mount Kenya Forest Ecosystem (Fig. 1) is a crucial ecological and hydrological gradient in central Kenya, located between 0.0°–0.3°S and 37.0°–37.5°E. The ecosystem supports diverse flora and fauna, including *Juniperus procera*, *Podocarpus falcatus*, and the endemic Mount Kenya mole shrew (*Surdisorex polulus*) (Hamerlynck et al., 2010; Pellikka et al., 2018). The mountain exhibits a distinct altitudinal gradient, transitioning from montane forests and bamboo zones to afro-alpine moorlands (Pellikka et al., 2018). Mount Kenya Forest plays a critical role in climate regulation, carbon sequestration, and water catchment functions, feeding major rivers such as the Tana and Ewaso Nyiro (Kenya Forest Service, 2010; Mwangi et al., 2020). The study area experiences varied temperatures ranging from about 5 °C in the upper zones, above 3000 m asl to 25 °C in the lower zones, with an average temperature reduction of about 0.6 °C for every 100 m increase in altitude. The dominant soils of the MKFE include Nitisols, Ferralsols, Andosols, Histosols, and Acrisols (Muchena & Gachene, 1988).

However, the MKFE is increasingly exposed to climate-induced stressors such as prolonged droughts, rising temperatures, and erratic precipitation patterns, which accelerate forest degradation, alter hydrological cycles, and reduce the resilience of endemic species. These shifts not only compromise ecosystem stability but also threaten downstream communities that rely on the forest for water, agriculture, and energy production.

Mount Kenya was selected as the study area due to its ecological and hydrological significance, serving as a critical biodiversity hotspot and one of Kenya’s major water towers (Jia et al., 2024). Its diverse vegetation zones provide an ideal setting for assessing climate-induced forest dynamics. Moreover, Mount Kenya has experienced notable climate variability and anthropogenic pressures, making it a suitable case study for evaluating climate-vegetation interactions.

### Data and sources

For this study, Landsat 7 ETM + (2000, 2010) and Landsat 8 OLI/TIRS (2020) imagery were utilized to assess vegetation dynamics in the MKFE. The data were accessed and processed using the Google Earth Engine (GEE) platform, which provides a preprocessed archive of petabytes of satellite images and analytical capabilities for exploring the data (Gorelick et al., 2017). To ensure consistency across datasets, one mosaicked image per year was created by compositing cloud-free scenes from the respective satellite missions. The following vegetation indices were derived to assess forest health: NDVI, EVI, and SAVI: Near-infrared (NIR) and red bands while BSI: Red, NIR, shortwave infrared (SWIR), and blue bands. Different indices were computed using Eqs. 1, 2, 3, and 4.

1 $$\text{NDVI}=\frac{\left(\text{NIR}-\text{RED}\right)}{(\text{NIR}+\text{RED})}$$

2 $$\text{EVI}=\text{G}* \frac{(\text{NIR}+\text{RED})}{\left(\text{NIR}+\text{C}1*\text{RED}-\text{C}2*\text{BLUE}+\text{L}\right)}$$

In this study, a typical value of L = 0.5 was used.

3 $$\text{SAVI}= \frac{\left(\text{NIR}-\text{RED}\right)}{\left(\text{NIR}+\text{RED}+\text{L}\right)}*\left(1+\text{L}\right)$$

where G is the gain factor (usually set to 2.5), L is the canopy background adjustment value (usually set to 10000), C1 is the coefficient for the red band (typically set to 6), and C2 is the coefficient for the Blue band (typically set to 7.5).

4 $$\text{BSI}= \frac{\left(\text{SWIR}+\text{RED}\right)-(\text{NIR}+\text{BLUE})}{\left(\text{SWIR}+\text{RED}\right)+(\text{NIR}+\text{BLUE})}$$

These indices were used to analyze vegetation trends and detect potential land degradation across the study period. Landsat 7 images were processed at a 30-m spatial resolution, with cloud masking applied to minimize atmospheric interference. Landsat 8, with a 30-m spatial resolution and 12-bit radiometric resolution, provided greater sensitivity to subtle vegetation changes in 2020.

The selected vegetation indices; NDVI, EVI, SAVI, and BSI were chosen based on their established effectiveness in monitoring vegetation conditions across diverse ecological gradients, particularly in montane and tropical forest ecosystems. NDVI, which uses red and near-infrared reflectance, provides a robust measure of overall vegetation greenness but can saturate in dense canopies such as those found in MKFE. EVI, which incorporates the blue band and corrects for atmospheric distortions and canopy background, is particularly suited for high-biomass, humid environments like MKFE’s upper montane zones. SAVI adjusts for soil brightness and is effective in areas with sparse vegetation or partial canopy cover, such as transitional zones undergoing degradation. BSI, by contrast, is useful in detecting bare soil exposure and vegetation loss, making it valuable for identifying degradation hot spots in lower montane zones. The combination of these indices enables a comprehensive assessment of vegetation health across the altitudinal and disturbance gradients present in MKFE.

To analyze climate impacts on vegetation, climate data were sourced from the CHIRPS and ERA5 datasets, selected for their spatial resolution, temporal coverage, and suitability for ecological modeling in data-scarce regions. CHIRPS (Climate Hazards Group InfraRed Precipitation with Station data) provides high-resolution gridded precipitation data (0.05° or ~ 5 km), merging satellite imagery with in situ station data, which makes it particularly useful for capturing localized rainfall variability across MKFE’s complex topography. ERA5, developed by the European Centre for Medium-Range Weather Forecasts (ECMWF), is a state-of-the-art atmospheric reanalysis dataset offering hourly estimates of a large number of climate variables at a ~ 31 km resolution. It provides consistent temperature and humidity profiles across elevations, making it well-suited to MKFE’s altitudinal and climatic gradients. These datasets were chosen because they allow for the reliable estimation of extreme temperature and precipitation indices, which are critical for understanding climate stress across different vegetation zones in the ecosystem. These datasets were processed using GEE.

The extracted climate variables included maximum precipitation (MaxP) and minimum precipitation, number of extreme rainfall days (> 10 mm, > 20 mm), squared maximum precipitation (MaxP^2^) and squared maximum temperature (MaxT^2^). These variables were calculated for 2000, 2010, and 2020 to align with the vegetation index analysis. Despite their strengths, both CHIRPS and ERA5 datasets have inherent limitations. CHIRPS, while enhanced by station data, may still underrepresent extreme rainfall in regions with sparse ground observations, particularly in high-elevation zones of the MKFE (Funk et al., 2015). ERA5, being a reanalysis product with coarser spatial resolution (~ 31 km), may smooth out fine-scale microclimatic variations critical to upper montane forest dynamics (Hersbach et al., 2020). However, these limitations were mitigated through the integration of multi-decadal data and correlation-based model calibration, ensuring that the derived climate indices remained robust for ecosystem-level analysis and prediction.

**Fig. 2:**
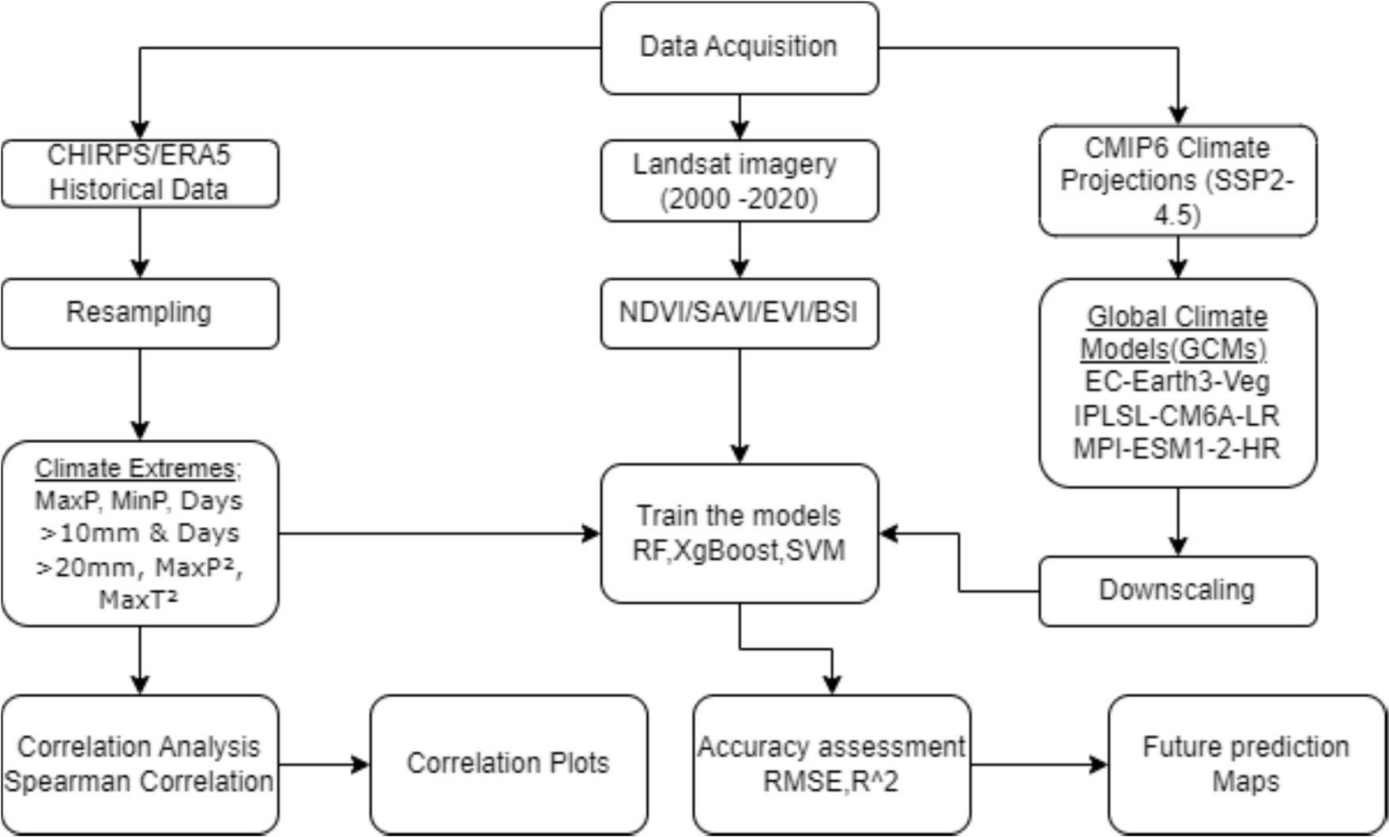
Overall study methodology workflow

Future climate conditions were analyzed using CMIP6 downscaled projections under the SSP2- 4.5 scenarios, representing a moderate emissions pathway. The study incorporated three General Circulation Models (GCMs): MPI-ESM1-2-HR – robust in simulating temperature and precipitation trends, IPSL-CM6 A-LR – effective for capturing precipitation extremes and EC-Earth3-Veg – incorporates land-use changes and vegetation dynamics. Climate variables were extracted for the 2021–2040 period, ensuring long-term climate impact assessments and a reasonable period to guide policy in the upcoming decades. All spatial analyses and modelling were conducted using QGIS, R, and Google Earth Engine (GEE). Key preprocessing steps included cloud masking for Landsat images, resampling climate datasets and vegetation indices to 10 m resolution for consistency and correlation analysis to assess the relationship between climate extremes and vegetation indices. This integrated approach provided a robust framework for assessing the impacts of climate change on the MKFE. Figure 2 summarizes the workflow followed in the study.

### Climate extremes computation

Climate extremes were derived using R libraries (*terra*, *dplyr*). Computed variables included: MaxP identifying extreme rainfall events, minimum precipitation representing drought severity, days with precipitation greater than 10 mm (> 10 mm) and days with precipitation greater than 20 mm (> 20 mm) counting moderate to heavy rainfall events, MaxP^2^ capturing cumulative impacts of extreme rainfall and MaxT^2^ quantifying extreme temperature events​. These metrics were analyzed to assess vegetation-climate interactions​.

### Machine learning analysis

Three machine learning models were applied: Random Forest (RF), extreme Gradient Boosting (XGBoost), and Support Vector Machines (SVM) models. The three selected models are widely used machine learning models for solving classification and regression problems. The models possess inherent strengths and limitations that apply across the model framework and are specific to each model. All the models are robust for handling data and modelling non-linear relationships between variables, with the SVM model requiring non-linear separable data for handling non-linear relationships. They are non-parametric models, which means they are “assumption-free” regarding data distributions. Each model also has inherent characteristics that have been evaluated to contribute to its high performance. For instance, the RF model is versatile for handling large datasets. The model is also sensitive to outliers and overfitting (Breiman, 2001). The XGBoost model handles large datasets, non-linear relationships and feature interactions (Chen & Guestrin, 2016), while SVM is suitable for high-dimensional data (Cortes & Vapnik, 1995).

The machine learning models underwent hyperparameter tuning to optimize their performance. The Random Forest (RF) model was optimized by varying the number of decision trees and the number of features considered by each tree when splitting a node. The optimal mtry value and the number of trees were selected based on cross-validation performance. The XGBoost model has numerous parameters, making it a complex model. Additionally, it requires hyperparameters to mitigate the risk of overfitting and reduce prediction variability. The XGBoost model underwent a more extensive hyperparameter tuning process using a cross-validated grid search. The hyperparameters tuned include n_estimators: 300, 400, 500 Eta: 0.01, 0.1, 0.3, max_depth: 3, 5, 9, min_child_weight: 1, 3, 5, subsample: 0.8, 1.0 and colsample_bytree: 0.7, 1.0, gamma: 0, 0.1, 0.2. The optimal values for the parameters were selected as 500, 0.3, 5, 3, 0.8, 1, and 0.1, respectively. The Support Vector Machine (SVM) model was trained using a radial basis function (RBF) kernel and tuned using a grid search over: sigma: 0.001, 0.01, 0.1, 1, and C: 0.1, 1, 10, 100. The optimal hyperparameters were selected based on fivefold cross-validation performance, ensuring a balance between model complexity and generalization. The effectiveness of each model was assessed using two key metrics: the coefficient of determination (*R*^2^) and the root mean squared error (RMSE).

The Random Forest (RF) model with the SAVI vegetation index was chosen as the primary model for further analysis due to its strong performance relative to other machine learning models and its comparability to the SLM benchmark. Key justifications include RF-SAVI achieved *R*^2^ = 0.63 and RMSE = 0.06, outperforming other machine learning models in predictive accuracy. Compared to the SLM model (*R*^2^ = 0.83, RMSE = 0.04), RF-SAVI exhibits strong predictive capability, offering the advantages of non-parametric modeling that allows for capturing complex, nonlinear relationships in the data. The improvement of RF over other machine learning models (XGBoost and SVM) suggests that ensemble-based approaches are more robust in capturing spatial variation in vegetation indices.

The models were trained using NDVI, EVI, SAVI, and BSI, which were dependent variables and climate extremes (MaxP, MaxP^2^, MaxT^2^), which were independent variables. The reason for selecting MaxP, MaxP^2^, and MaxT^2^ as the independent variables rather than using all Climate Extreme Indices (CEIs) was based on a correlation analysis conducted prior to model training. The correlation analysis was performed to understand the relationships between vegetation indices (EVI, NDVI, SAVI, BSI) and climate extremes (MaxP, MinP, Days > 10 mm, days > 20 mm, MaxP^2^, MaxT^2^). The analysis revealed that MaxP, MaxP^2^, and MaxT^2^ showed the strongest correlations with vegetation indices, meaning they had the most influence on predicting vegetation conditions​.

Including all CEIs could lead to multicollinearity, where highly correlated variables provide redundant information. By selecting only MaxP, MaxP^2^, and MaxT^2^, the model focuses on the most relevant predictors, ensuring efficiency and avoiding overfitting. The variables MinP, days > 10 mm, and days > 20 mm showed lower correlation coefficients with vegetation indices, making them less influential in predictive modelling. Including weakly correlated variables could introduce noise, reducing model accuracy. Data from 2000, 2010, and 2020 were split into 80% training and 20% testing sets. fivefold cross-validation was applied to prevent overfitting. Models were assessed using Root Mean Squared Error (RMSE) – measures prediction accuracy and coefficient of determination (*R*^2^) – Explains variance in vegetation indices. The trained models were saved as RDS files for future applications.

### Predictive modeling

Predictive modelling was conducted to assess future vegetation trends under climate change scenarios. The dependent variables were vegetation indices representing forest health, while the independent variables included climate extremes derived from CMIP6 SSP2-4.5 projections, Squared Maximum Precipitation (MaxP^2^), and Squared Maximum Temperature (MaxT^2^). The three machine learning models were applied to predict future vegetation conditions. The models were trained on historical vegetation indices (2000, 2010, 2020) and applied to future climate projections (2021–2040) using three General Circulation Models (GCMs): EC-Earth3-Veg, IPSL-CM6 A-LR, and MPI-ESM1-2-HR. The years 2000, 2010, and 2020 were selected to capture long-term forest change trends in the two-decade period. These intervals align with major climate assessment periods, allowing for the evaluation of gradual ecosystem shifts rather than short-term fluctuations. Moreover, the selection coincides with significant climate events, such as prolonged droughts and temperature anomalies in East Africa, which have influenced forest health and structure. This approach ensures a robust analysis of climate-induced changes rather than transient variations. The model with the highest accuracy was selected to model the vulnerability of MKFE to future climatic conditions.

Random Forest (RF) was selected as the primary regression model due to its robustness in handling high-dimensional, non-linear datasets common in climate-ecological studies. Compared to Support Vector Machines (SVM), which require complex kernel tuning, and neural networks, which are data-intensive and susceptible to overfitting, RF offers high predictive accuracy with minimal parameter tuning. It is particularly advantageous in ecological modeling as it can manage noisy data, capture non-linear interactions between vegetation indices and climate extremes, and provide feature importance metrics to interpret predictor influence. These capabilities made RF especially suitable for modeling the complex climate–vegetation relationships in the heterogeneous MKFE landscape.

### Vulnerability mapping

Vulnerability mapping was performed by comparing predicted EVI values (2021–2040) with baseline EVI (2020) to detect areas of vegetation loss, stability, or improvement. The index revealed robust estimates for characterizing forest ecosystem conditions relative to other indices. Therefore, to establish the vulnerability, the difference between the index value under the baseline and the predicted EVI was considered. Vulnerability thresholds were defined as loss (EVI decrease <  − 0.05), stable (− 0.05 ≤ EVI change ≤ 0.05), and gain (EVI increase > 0.05). Vulnerability was classified using R: Raster values were categorized into three vulnerability classes (loss, stable, gain) using the cut () function. Spatial vulnerability maps were generated using the raster (Hijmans & van Etten, 2014) and ggplot2 (Wickham & Sievert, 2009) libraries.

## Results

### Vegetation dynamics across indices

The analysis of vegetation indices revealed notable temporal and spatial variations in vegetation health across Mount Kenya. Between 2000 and 2010, vegetation indices exhibited slight improvements, largely attributed to favorable climatic conditions and conservation initiatives. However, by 2020, a significant decline was observed, indicating the adverse impacts of deforestation, agricultural expansion, and climate-induced stressors.

### NDVI, EVI, SAVI, and BSI trends

**Fig. 3:**
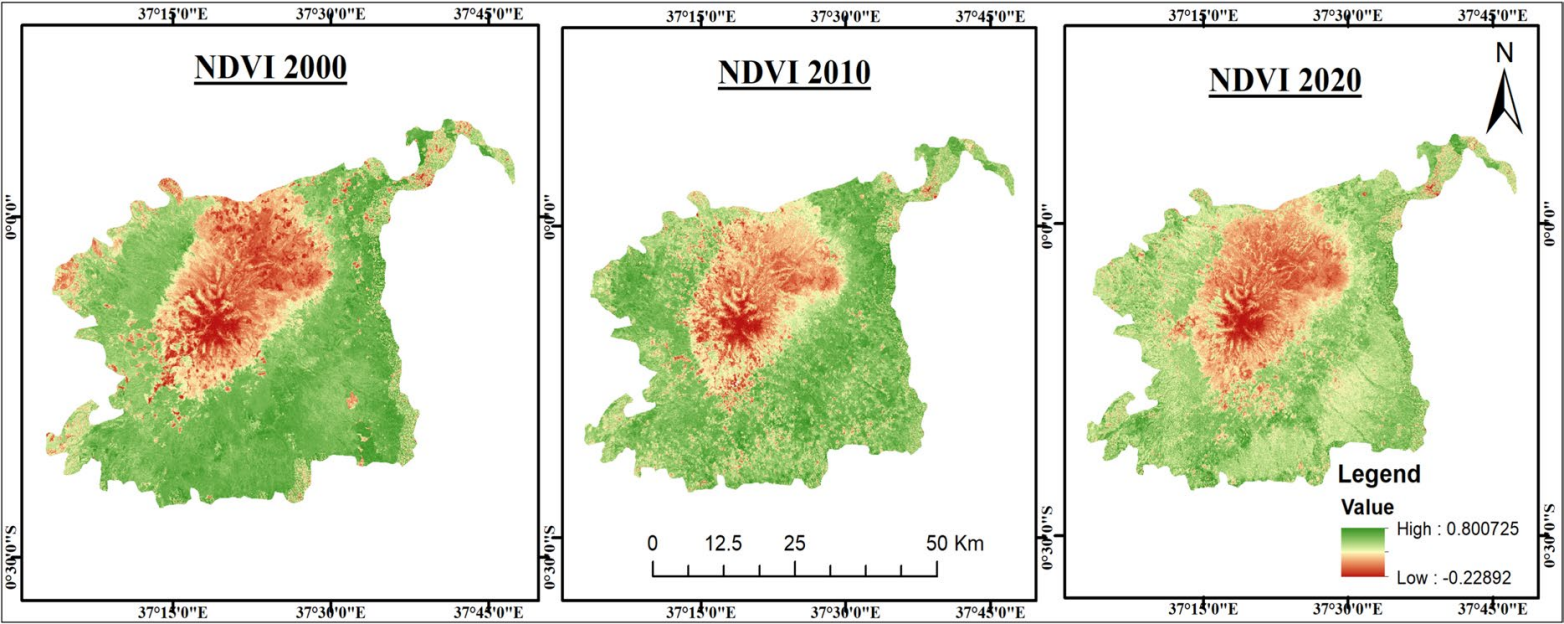
Normalized Difference Vegetation Index (NDVI) maps

NDVI results indicate a sharp decline in vegetation health over time (Fig. 3). Between 2000 and 2010, mean NDVI increased slightly from 0.47 to 0.48, suggesting stable or improving vegetation conditions. However, by 2020, the mean dropped to 0.26 (a 45.4% decline), accompanied by a reduction in maximum NDVI from 0.73 (2000) to 0.44 (2020). Spatially, NDVI > 0.7 areas disappeared entirely by 2020, and the emergence of negative values (− 0.02) reflects bare soil or degraded land. These results suggest the combined impacts of deforestation, land-use changes, and climate stressors. The decline in dense vegetation (NDVI > 0.7) highlights the increasing vulnerability of the ecosystem to degradation.

**Fig. 4:**
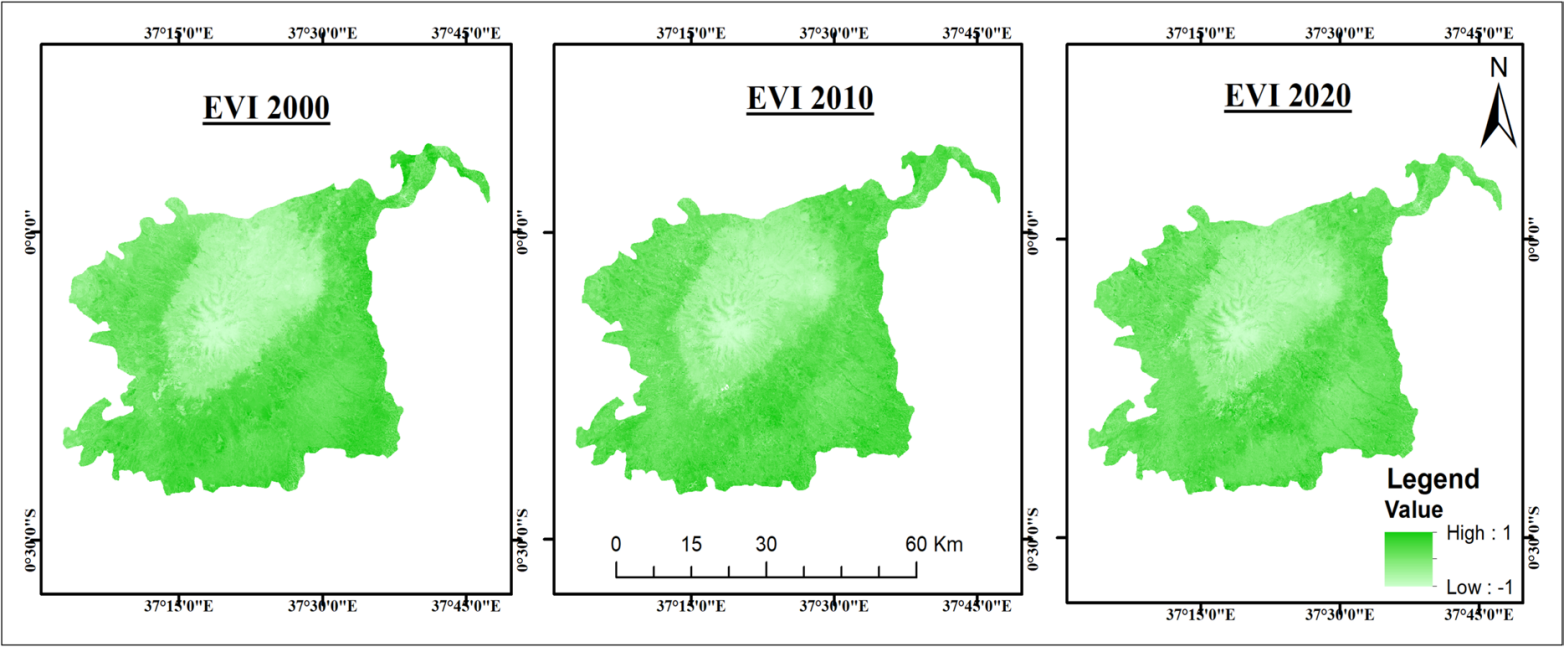
Enhanced Vegetation Index (EVI) maps

**Fig. 5:**
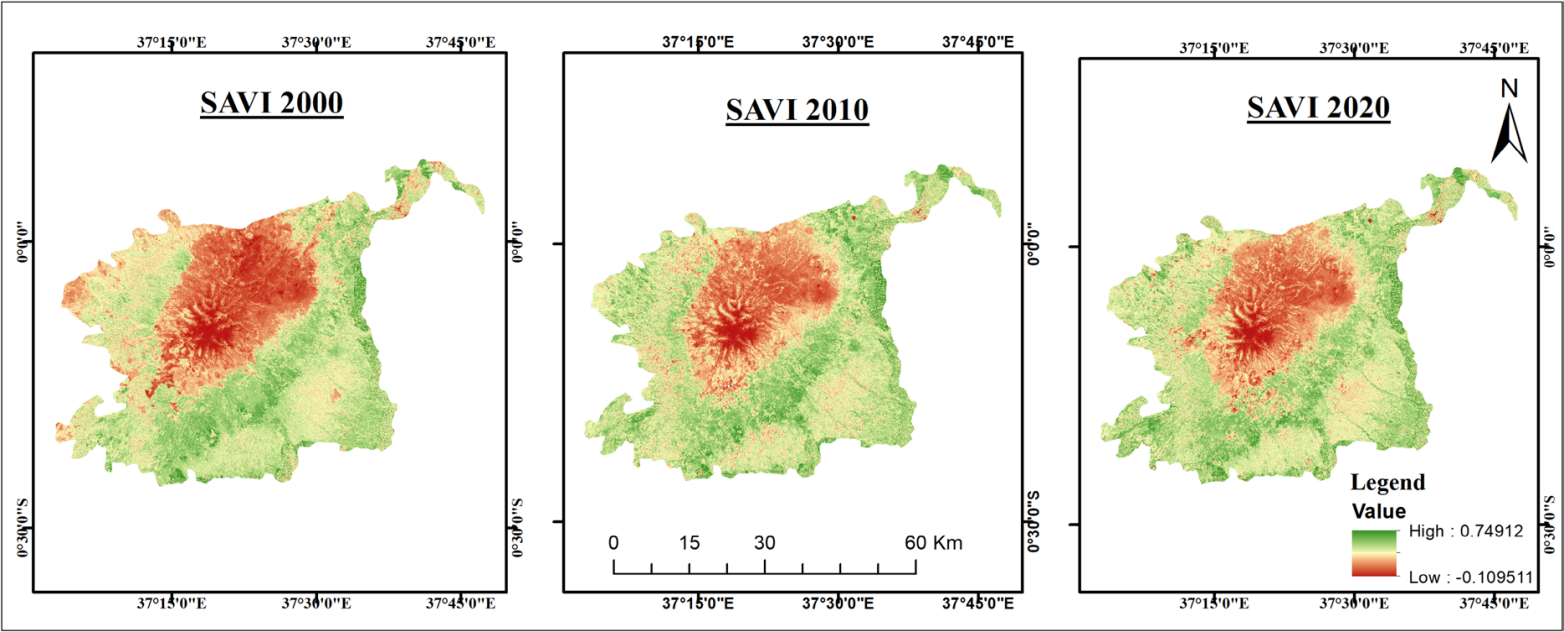
Soil-Adjusted Vegetation Index (SAVI) maps

**Fig. 6:**
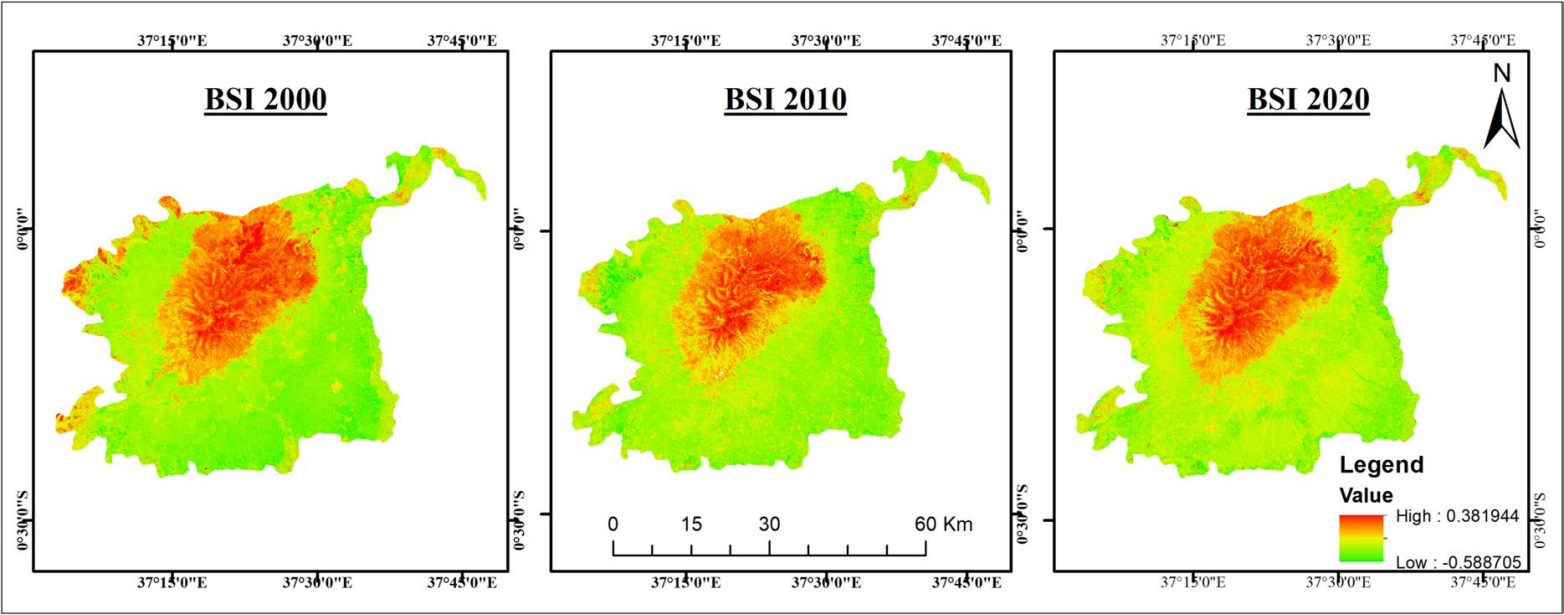
Bare Soil Index (BSI) maps

EVI and SAVI followed similar trends as NDVI, with EVI declining by 13.3% and SAVI by 8.3% from 2010 to 2020 (Figs. 4 and 5). BSI increased by 55.1%, indicating a major expansion of bare soil areas, largely due to vegetation loss from deforestation and climate variability (Fig. 6).

### Correlation analysis

**Fig. 7:**
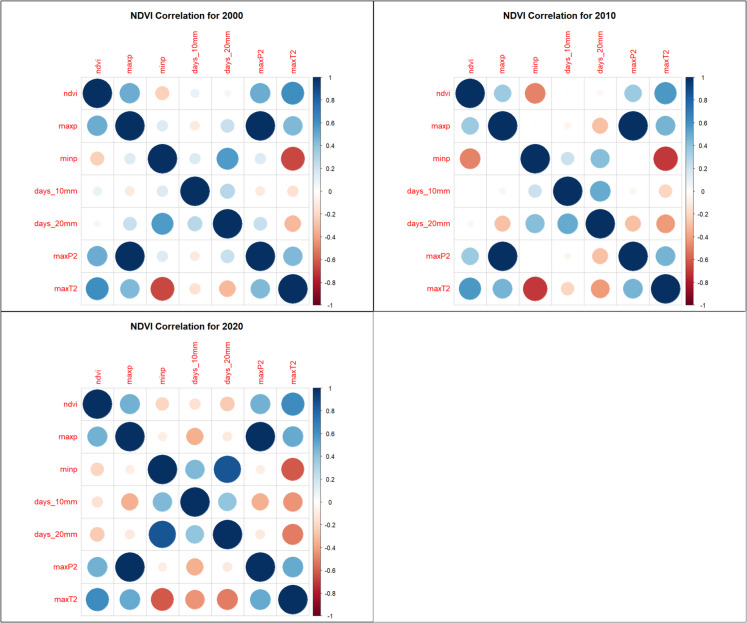
Normalized Difference Vegetation Index (NDVI) correlation heatmaps

**Fig. 8:**
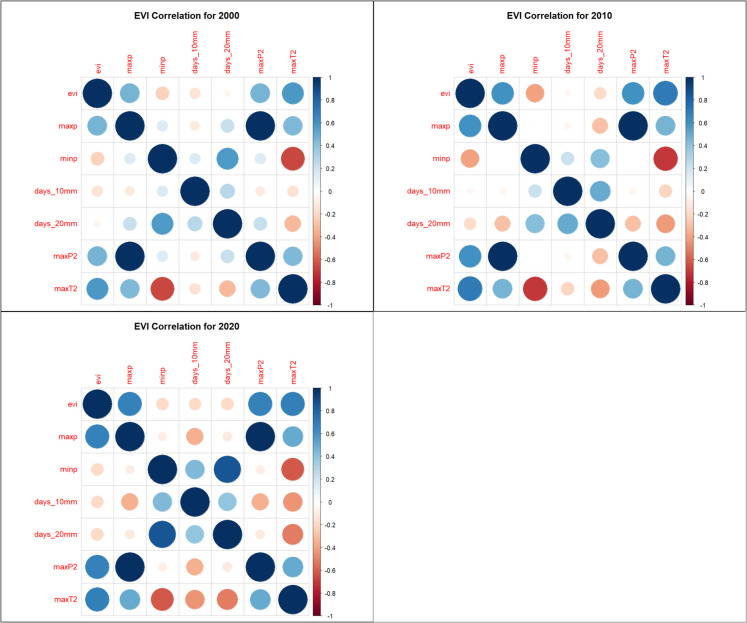
Enhanced Vegetation Index (EVI) correlations heatmaps

**Fig. 9:**
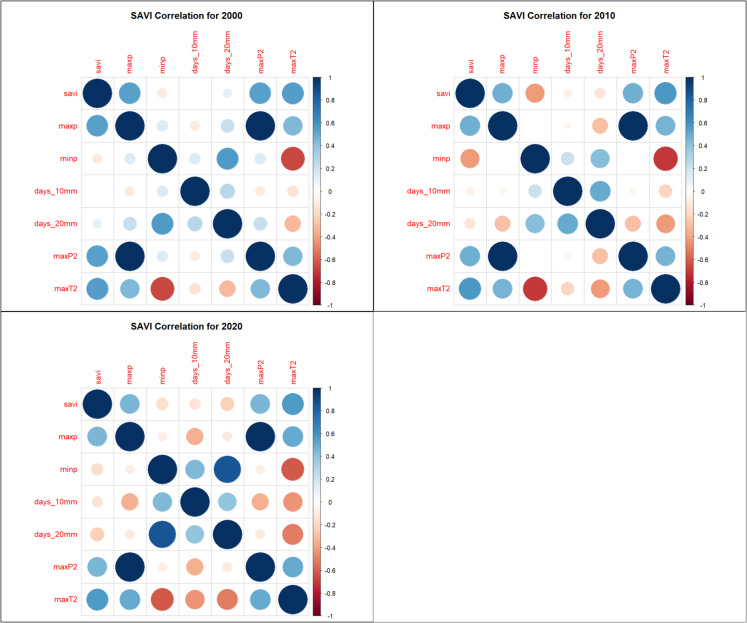
Soil-Adjusted Vegetation Index (SAVI) correlations heatmaps

**Fig. 10:**
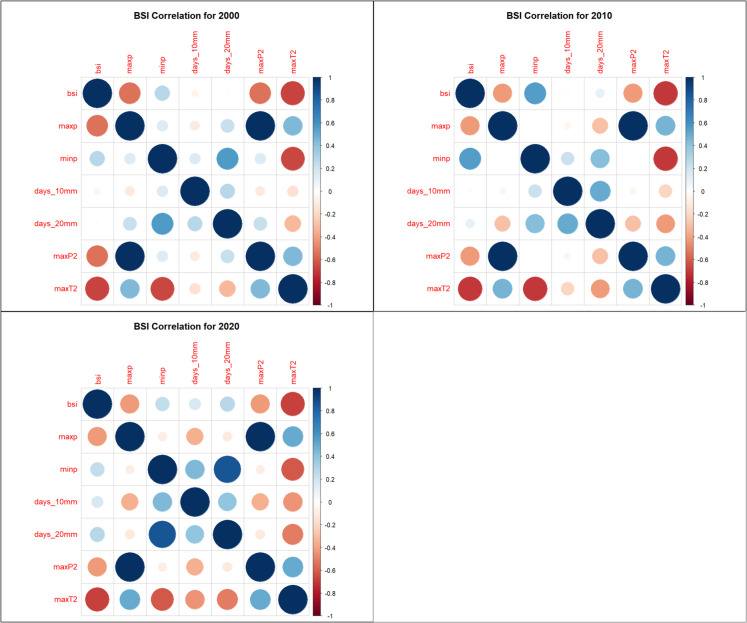
Bare Soil Index (BSI) correlation heatmaps

Spearman rank correlation analysis identified strong relationships between vegetation indices and climate variables: NDVI, SAVI, and EVI had strong positive correlations with MaxP, MaxP^2^, and MaxT^2^, suggesting that precipitation and moderate temperature extremes favor vegetation growth (Figs. 7, 8, and 9). NDVI, SAVI, and EVI were strongly negatively correlated with MinP, indicating that prolonged dry conditions reduce vegetation health (Figs. 7, 8, and 9). BSI had a strong negative correlation with MaxT^2^ and MaxP but was positively correlated with MinP, reinforcing its inverse relationship with vegetation indices (Fig. 10).

### Model training and validation performance

The RF, XGBoost, and SVM models were trained and validated using climate data as explanatory variables. Performance metrics revealed: RF performed best for EVI-based vulnerability mapping (*R*^2^ = 0.82, RMSE = 0.07. SVM achieved high accuracy for the NDVI variable (*R*^2^ = 0.69, RMSE = 0.05) and BSI (*R*^2^ = 0.75, RMSE = 0.04). XGBoost: Performed well but did not consistently outperform RF and SVM across all indices. The validation metrics for the selected vegetation indices and machine learning models are displayed in Table 1.

**Table 1:** Validation metrics for vegetation indices and machine learning models

Model	Vegetation Index	R^2^	RMSE
Random Forest	EVI	0.82	0.07
	NDVI	0.64	0.05
	SAVI	0.58	0.06
	BSI	0.69	0.04
XGBoost	EVI	0.62	0.08
	NDVI	0.69	0.07
	SAVI	0.66	0.07
	BSI	0.61	0.07
Support Vector Machine	EVI	0.57	0.08
	NDVI	0.68	0.04
	SAVI	0.59	0.05
	BSI	0.74	0.04

### Future vegetation predictions under climate change

**Fig. 11:**
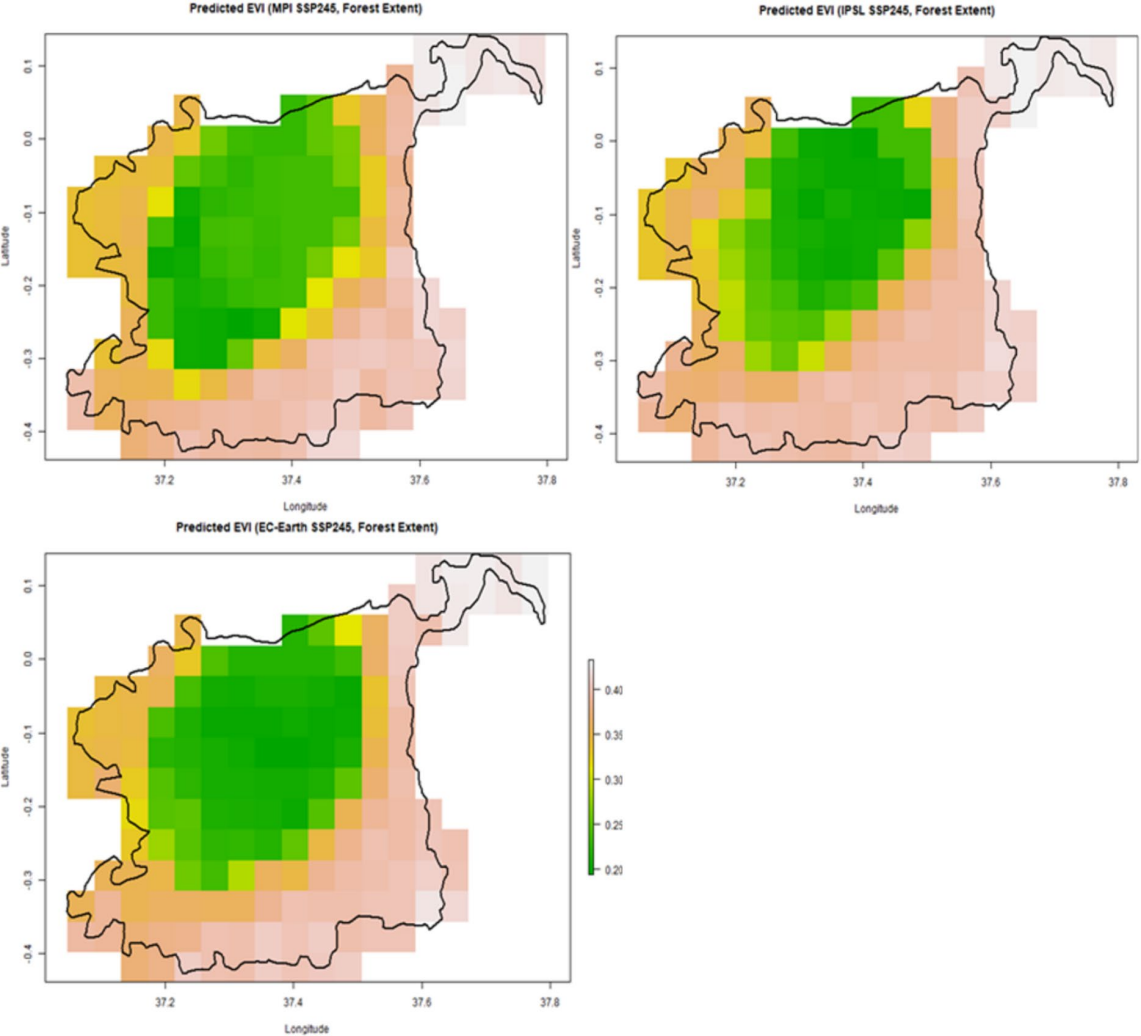
Predicted EVI maps of Mount Kenya Forest Ecosystem from MPI-ESM1-2-HR, IPSL-CM6 A-LR, and EC-Earth3-Veg climate models for the period 2021–2040 under SSP2-4.5 scenario

Using CMIP6 SSP2-4.5 projections, the models predicted vegetation trends for 2021–2040: EC-Earth3-Veg Model: Stable EVI values in higher altitudes but declines in mid- and lower-elevation zones. IPSL-CM6 A-LR Model: Largest EVI reductions in mid-elevation regions, linked to precipitation variability and drought periods. MPI-ESM1-2-HR Model: Moderate declines in lower elevations but stable vegetation in higher-altitude zones. Future vegetation predictions for climate change are displayed in Fig. 11.

### Spatial variability in vegetation vulnerability under future climate scenarios

**Fig. 12:**
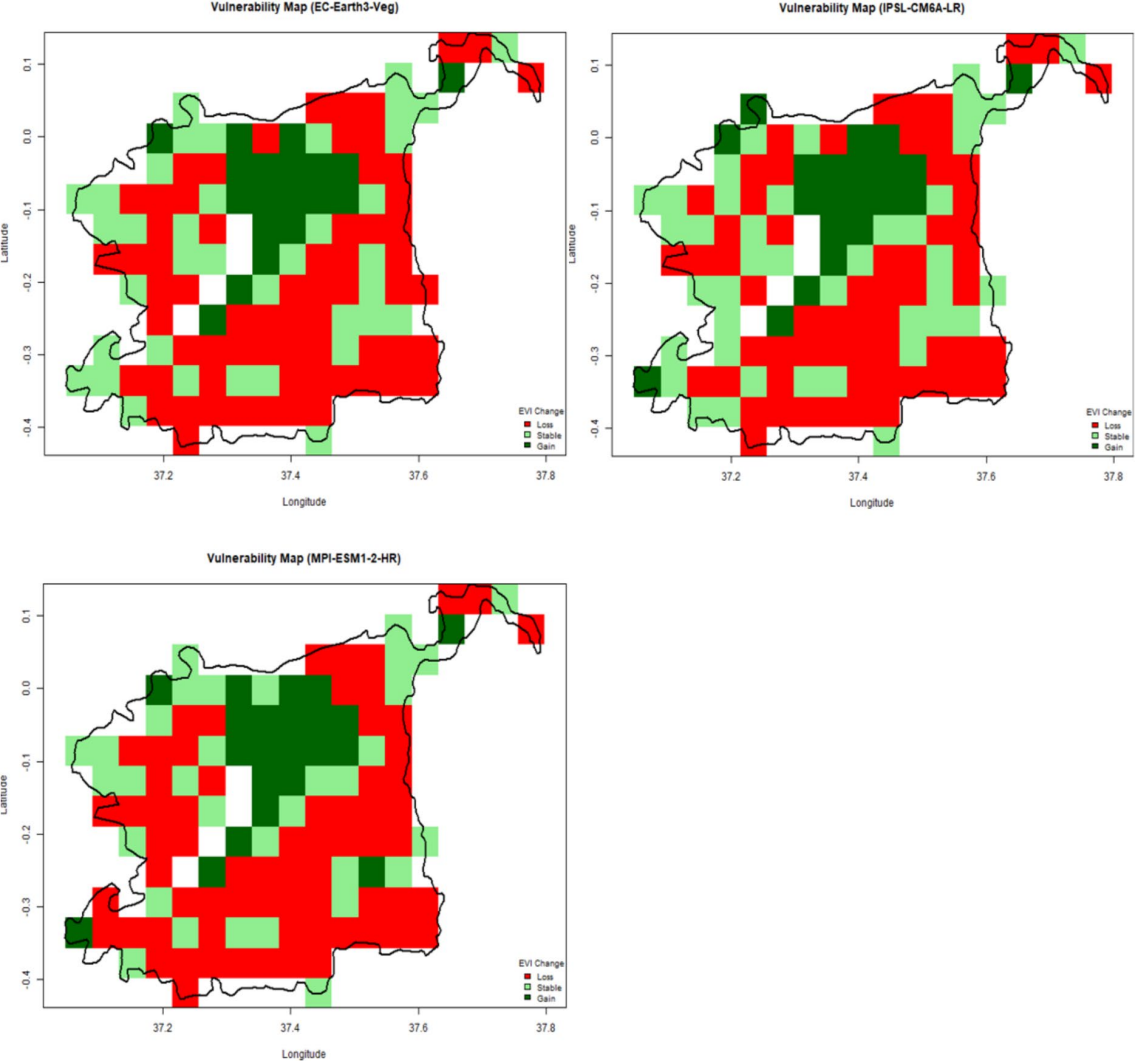
Vulnerability maps of Mount Kenya’s forest ecosystem analyzed from EC-Earth, IPSL-CM6 A-LR, MPI-ESM1-2-HR models under the SSP2-4.5 scenario

**Fig. 13:**
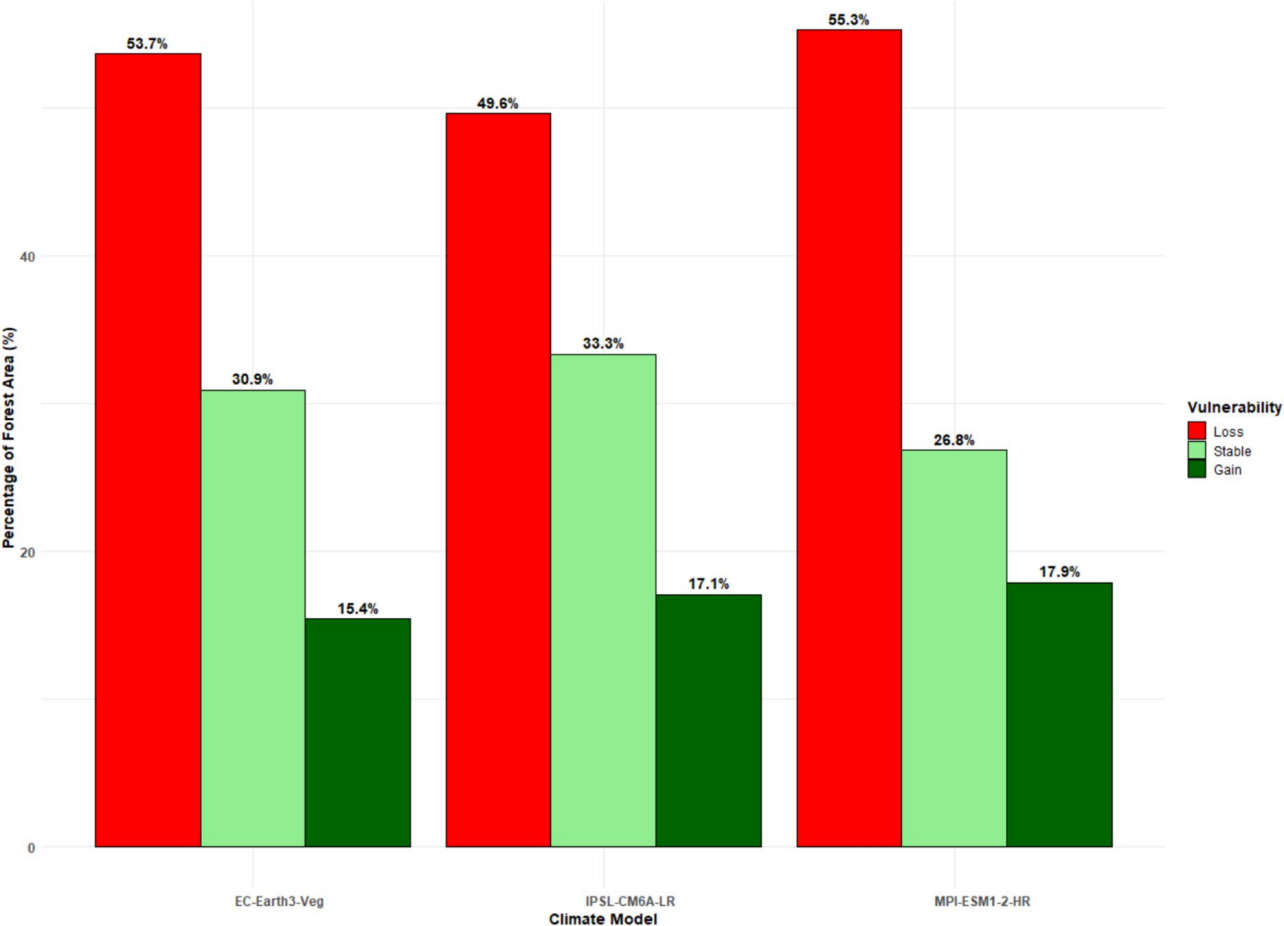
Percentage of forest change in Mount Kenya’s forest under different General Circulation Models (GCMs)

Vulnerability maps were created by comparing projected EVI values (2021–2040) to baseline EVI (2020): Lower Montane Areas: High vulnerability due to deforestation and increased climate stress. Upper Montane Areas: Some vegetation recovery was observed, likely due to favorable microclimates and conservation efforts. Central Zones: Minimal vegetation change, indicating relative stability. Figure 12 shows the spatial variability in vegetation vulnerability under future climate scenarios, whereas Fig. 13 shows the proportions of the different vulnerability classes across the analyzed GCMs. The models show marked similarities in terms of the characterization of future vulnerabilities across the MKFE. For instance, the central regions of the ecosystem reveal low vulnerability and indicate improved conditions in future. The middle areas reveal a stable trend with conditions likely to remain unchanged under future climates. However, the outer region reveals loss or depreciated vegetation conditions, showing that these areas are likely to experience low vegetation conditions in future.

Model projections indicate an even more substantial 49–55% decline in Enhanced Vegetation Index (EVI) values across forested zones by 2040. This magnitude of decline suggests not just seasonal stress, but long-term structural changes in forest cover, including reduced canopy density, lower photosynthetic productivity, and declining ecosystem resilience. Such changes may compromise the MKFE’s ability to provide critical ecosystem services such as carbon storage, habitat connectivity, and hydrological regulation.

Lower montane zones emerge as the most vulnerable regions in the projections. Their vulnerability is driven by both ecological and anthropogenic factors. Ecologically, these zones lie at a transitional altitude where precipitation variability, soil instability, and temperature extremes are more pronounced. They’re also subject to more intense human activity, such as land-use pressure from farming, settlement, and logging. Unlike the upper montane zones, which benefit from cooler microclimates and reduced human activity, lower montane areas lack buffering mechanisms, making them especially sensitive to both climate extremes and human encroachment. The combined effect heightens the risk of forest degradation, biodiversity loss, and ecosystem service disruption in these zones.

## Discussion

The present study analyzed the prevailing vegetation conditions of the Mount Kenya Forest Ecosystem and predicted its vulnerability under future climate scenarios. The study’s results reveal distinct temporal and spatial patterns in vegetation health across Mount Kenya. Between 2000 and 2010, there was a significant improvement in vegetation indices attributed to favorable precipitation conditions and conservation efforts. However, from 2010 to 2020, a notable decline was observed: NDVI dropped by 45.4%, EVI decreased by 13.3%, SAVI declined by 8.3%, and BSI increased by 55.1%, indicating widespread vegetation loss and bare soil exposure. This observed decline correlates with increased deforestation, agricultural expansion, and the impact of climate extremes such as erratic rainfall and rising temperatures. These findings align with previous research in montane ecosystems, where anthropogenic pressures and climate variability have been identified as key contributors to vegetation degradation (Rotich & Ojwang, 2021; Yu et al., 2024). Similar trends have been documented in highland forests of East Africa, where NDVI and EVI declines were directly linked to land-use changes and shifting precipitation patterns (Abera et al., 2022). Some parts of the East African highlands have experienced severe vegetation loss due to prolonged droughts and increased human activities, leading to the fragmentation of natural forests and the expansion of bare lands (Matongera et al., 2021; Rotich et al., 2022, 2025).

The integration of remote sensing and vegetation indices has provided insights into vegetation cover dynamics and land degradation processes in East Africa. Studies using MODIS and Landsat data have shown that NDVI and SAVI declines are highly correlated with land cover conversion from forest to agriculture and urban expansion (Hamunyela et al., 2022). Additionally, climate-induced shifts in temperature and precipitation patterns have further accelerated the decline of vegetation health in montane regions (Wang et al., 2025).

The increase in BSI values indicates an increase in exposed soil and reduced vegetation cover, a trend also observed in studies focusing on rangeland ecosystems in Ethiopia and Kenya (Tiruneh et al., 2022). In such ecosystems, reduced vegetation has been linked to overgrazing, unsustainable land-use practices, and recurrent drought conditions (Abera et al., 2022; Kipkulei et al., 2022). Moreover, research by Sierra-Morales et al. (2021) reveals that montane vegetation is highly sensitive to temperature shifts, which can lead to altitudinal migration of vegetation zones and disruptions in ecological stability. Such ecosystem shifts are already being observed in East Africa, where woody cover is declining due to increased temperatures and prolonged dry seasons (Senanayake et al., 2022).

The correlation analysis in this study revealed significant relationships between climate variables and vegetation indices in the MKFE. MaxP and MaxP^2^ showed strong positive correlations with NDVI, EVI, and SAVI, indicating that extreme precipitation events enhance vegetation growth. MaxT^2^ was also positively associated with vegetation indices, suggesting that moderate warming enhances plant productivity in the study area. MinP was negatively correlated with vegetation indices, indicating that drought conditions negatively impact vegetation health. BSI was negatively correlated with MaxT^2^ and MaxP, reflecting an increase in bare soil exposure under extreme climatic conditions. These results are consistent with global research on the impacts of climate variability on vegetation indices. In the East African highlands, NDVI and EVI have shown strong seasonal dependencies on rainfall. During wetter periods, vegetation indices peak, while during dry seasons, significant declines are recorded (Pandit et al., 2024). In tropical montane cloud forests, vegetation indices such as NDVI and SAVI were found to be highly sensitive to changes in precipitation regimes, affecting forest health and structure (Yu et al., 2024).

The vulnerability mapping analysis identified distinct spatial patterns of vegetation change across Mount Kenya. Lower Montane Areas (high vulnerability): Marked by significant vegetation loss, driven by deforestation, agricultural expansion, and sensitivity to climate stressors. Upper Montane Areas (low vulnerability): Showed vegetation improvement, likely due to reduced human disturbance and favorable microclimatic conditions. Central Zones (moderate vulnerability): Exhibited minimal change, suggesting relative stability in vegetation health. The vulnerability maps emphasize the need for targeted conservation interventions. Similar studies in tropical montane forests highlight that lower-altitude zones are more vulnerable to climate extremes and land-use pressures, requiring immediate intervention (Li et al., 2022).

The study evaluated the predictive power of three machine learning models, RF, SVM and XGBoost and found the models to have high accuracy in evaluating drivers of forest conditions. Nonetheless, the RF model outperformed other models. These results support findings from similar studies, where RF consistently outperforms other models in predicting vegetation dynamics (Yu et al., 2024). However, these models, together with the climatic data integrated into the analysis, possess different uncertainties that could potentially affect the model results. The approach adopted in the present study, where multiple machine learning models are evaluated, is effective in analyzing the predictive performance and examining the vegetation-climate characteristics. The model performance was comparable across the models, with the RF model revealing the best performance. Also, the assessment of vegetation condition vulnerability using three GCMs allowed for the comparison of future climate impact across plausible climate futures. The multi-model approach is effective for assessing the vegetation conditions, given the varying climate conditions for different climate models.

Similar climate-induced forest degradation patterns have been observed in other montane ecosystems globally. Studies in the Eastern Himalayas (Jha et al., 2022) and the Andean forest along the Andes mountain range (Quinlan et al., 2025) report upslope migration of vegetation due to rising temperatures, mirroring the species redistribution trends observed in Mount Kenya. These shifts, driven by temperature increases and changing precipitation patterns, disrupt ecological gradients and threaten endemic biodiversity. Likewise, research in European alpine forests highlights increased drought stress and tree mortality, aligning with the moisture stress trends identified in this study (Gazol & Camarero, 2022). Additionally, the application of remote sensing and machine learning to assess forest vulnerability has been explored in temperate and tropical mountains (Singha et al., 2024), further reinforcing the effectiveness of this study’s approach. These comparisons indicate that montane forests worldwide are experiencing convergent climate vulnerability trends, emphasizing the need for integrated conservation strategies, predictive modeling, and transboundary collaboration to mitigate ecosystem risks.

The study highlights the importance of adaptive conservation strategies and their implications for forest management and conservation within the Mt. Kenya Forest Ecosystem. The ecosystem exemplifies one of the crucial natural resources in Kenya, which is of significant importance to key sectors such as forestry, water, and tourism. Furthermore, the ecosystem plays a significant role in carbon sequestration, thus contributing to climate change mitigation efforts. As climate change alters precipitation and temperature patterns, conservation efforts must be tailored to enhance ecosystem resilience and maintain biodiversity. Despite the robust estimates from the modelling approaches used in the present study, we highlight some limitations which can be used to improve findings for future research. One limitation is that we used optical remote sensing data for condition monitoring and analysis. The data is affected by cloud cover, which is particularly problematic in montane regions and might hinder the retrieval of accurate vegetation conditions.

To improve precision, future research should integrate multi-sensor approaches, combining Landsat’s temporal depth, Sentinel-2’s finer resolution, and radar-based data (e.g., Sentinel-1 SAR, ALOS PALSAR) to mitigate cloud interference and enhance forest change detection accuracy. Secondly, future studies should evaluate vegetation conditions using additional climate models to capture the entire range of plausible climate change scenarios. Future research should also focus on species-level ecological responses to specific climate extremes, such as heatwaves or prolonged droughts, particularly in vulnerable zones like the lower montane belt. This includes assessing shifts in the distribution and health of endemic or endangered species (e.g., the *Mount Kenya Bush Viper* or *Mountain Bongo*), as well as quantifying how reduced forest cover impacts key ecosystem services such as carbon storage and watershed regulation.

Kenya’s Forest Conservation and Management Act (2016) and the Climate Change Act (2016) provide a strong legal foundation for enforcing sustainable land-use practices through mechanisms such as community forest associations, mandatory forest management plans, and climate resilience integration into development planning. These frameworks support adaptive management by enabling local governance structures to implement afforestation initiatives using native and climate-resilient species such as *Juniperus procera*, *Podocarpus falcatus*, and *Hagenia abyssinica*, which are well-adapted to montane conditions and provide critical habitat and watershed functions. On the ground, sustainable land-use planning can be operationalized through zoned agroforestry, payment for ecosystem services (PES), and stricter regulation of encroachment and charcoal production. In the context of the Mount Kenya Forest Ecosystem (MKFE), policies such as the Participatory Forest Management (PFM) regulations and the National Climate Change Action Plan (NCCAP) can be leveraged to align conservation with livelihood needs, ensuring both ecological integrity and socio-economic sustainability.

## Conclusion

This study assessed the impact of climate change on Mount Kenya’s forests by analyzing vegetation dynamics and their correlations with climatic variables. Key findings include: The decline in vegetation health, particularly a 45.4% decline in normalized difference vegetation index (NDVI), accompanied by a 55.1% increase in bare soil index (BSI), underscores the compounded impacts of deforestation, agricultural expansion, and climate-induced stressors such as erratic rainfall and rising temperatures. This highlights the urgency of addressing deforestation drivers and climate adaptation strategies.

Vegetation indices NDVI, soil-adjusted vegetation index (SAVI), and enhanced vegetation index (EVI) showed strong positive correlations with maximum precipitation (MaxP), cumulative extreme precipitation (MaxP^2^), and maximum temperature squared (MaxT^2^). However, they were strongly negatively correlated with minimum precipitation. In contrast, BSI was negatively correlated with MaxT^2^ and MaxP but positively correlated with MinP, showcasing its inverse relationship to vegetation indices.

These relationships indicate that extreme precipitation and temperature variability are key drivers of forest dynamics in the Mount Kenya Forest Ecosystem (MKFE). Lower montane areas exhibited significant vegetation loss, making them more vulnerable to climate-induced degradation. In contrast, upper montane areas demonstrated vegetation improvement, while the central zones showed minimal changes, emphasizing spatial heterogeneity in climate impacts.

This research contributes to the understanding of climate-vegetation dynamics in montane ecosystems by highlighting the temporal trends of vegetation indices and their response to climatic variability, demonstrating the utility of advanced vegetation indices (e.g., EVI, SAVI, BSI) and climate extremes in vulnerability mapping, providing spatial insights into areas most vulnerable to vegetation degradation and aiding targeted conservation planning. These findings underscore the critical role of integrating climate projections, remote sensing, and vegetation indices in monitoring forest responses to climate change, offering actionable insights for forest management and policy interventions.

This study provides a scalable approach for integrating remote sensing, climate modeling, and machine learning to assess forest vulnerabilities under changing climate conditions. The insights gained can inform targeted conservation strategies, adaptive forest management, and climate risk mitigation policies. Future research should focus on refining prediction models by incorporating higher-resolution satellite imagery and additional climate scenarios to enhance accuracy. Moreover, expanding the analysis to other East African montane forests, such as the Aberdare Range and Mau Forest Complex, would provide a regional perspective on climate-induced ecosystem transformations. Strengthening collaborations between researchers, policymakers, and conservationists is crucial in developing proactive solutions for sustaining mountain forest ecosystems amid escalating climate change pressures.

The approach employed in this study is broadly transferable to other montane and forested ecosystems globally. Its core strength lies in integrating high-resolution remote sensing, climate extremes, and machine learning, allowing it to adapt to diverse ecological contexts. In tropical forests (e.g., Andes, Eastern Arc Mountains), similar vegetation indices and climate proxies can be used, though attention must be given to cloud cover limitations and terrain correction. In temperate regions, the methodology can be adapted using seasonally adjusted indices and phenology-based modeling. However, regional calibration is essential, especially in selecting appropriate vegetation indices, climate variables, and species-specific responses to stress like drought or heat. This adaptability makes the approach a valuable tool for scalable ecosystem risk assessments under climate change.

## Data Availability

No datasets were generated or analysed during the current study.

## Declarations

All authors have read, understood, and have complied as applicable with the statement on “Ethical responsibilities of Authors” as found in the Instructions for Authors.
